# Preoperative CT and inflammatory index–based risk scores for predicting short-term recurrence following resection of pancreatic ductal adenocarcinoma

**DOI:** 10.1186/s12893-026-03948-3

**Published:** 2026-07-01

**Authors:** Mohammad Masih Mansouri-Tehrani, Farhad Zamani, Hamed Iraji, Fahimeh Safarnezhad Tameshkel, Mohammad Mersad Mansouri Tehrani, Moein Ghasemi

**Affiliations:** 1 Department of Radiology, School of Medicine, Iran University of Medical Sciences, Tehran, Iran; 2 Gastrointestinal and Liver Disease Research Center, Iran University of Medical Sciences, Tehran, Iran; 3 Department of Radiology, Advanced Diagnostic and Interventional Radiology Research Center (ADIR), Tehran University of Medical Science, Tehran, Iran; 4 School of Medicine, Tehran University of Medical Sciences, Tehran, Iran

**Keywords:** Pancreatic ductal adenocarcinoma, Computed tomography, Risk stratification, Prognostic model, Recurrence

## Abstract

**Background:**

Pancreatic ductal adenocarcinoma (PDAC) carries a poor prognosis, with nearly half of resected patients relapsing within one year. Current National Comprehensive Cancer Network (NCCN) and American Joint Committee on Cancer (AJCC) staging systems inadequately reflect tumor biology. This study aimed to develop and validate preoperative risk scores integrating computed tomography (CT) features and systemic inflammatory markers to predict 12-month recurrence.

**Methods:**

In this two-center retrospective cohort, consecutive patients with histologically confirmed resectable/borderline PDAC who underwent pancreaticoduodenectomy (2017–2024) were included. Standardized preoperative multiphase CT and laboratory data within 28 days before surgery were reviewed. Quantitative and qualitative CT parameters and inflammatory indices were analyzed using multiple imputation and pathway-specific logistic regression for up-front surgery (US) and neoadjuvant therapy (NT) groups.

Model performance was evaluated by discrimination, calibration, and decision-curve analysis.

**Results:**

Of 544 resections, 133 met eligibility (US = 81; NT = 52). Final models retained tumor size, necrosis, fat stranding, and the C-reactive protein-to-albumin ratio (CAR) and neutrophil-to-lymphocyte ratio (NLR) for US, and tumor size, hepatic-artery contact ≥ 90°, CAR, NLR, and carcinoembryonic antigen (CEA) for NT. Development area under the curve (AUC) values were 0.84 (US) and 0.81 (NT), and test AUCs were 0.82 and 0.78, respectively, outperforming AJCC 8ᵗʰ staging (ΔAUC + 0.19 and + 0.20).

**Conclusions:**

Compact, pathway-specific preoperative scores combining CT descriptors and inflammatory indices improved prediction of early recurrence compared with AJCC staging, supporting their potential use for individualized surgical and perioperative decision-making.

**Supplementary Information:**

The online version contains supplementary material available at https://doi.org/10.1186/s12893-026-03948-3.

## Background

Pancreatic ductal adenocarcinoma (PDAC) remains one of the deadliest solid malignancies, and the incidence of pancreatic cancer continues to rise, projected to become the second leading cause of cancer-related deaths in the United States by 2040 [1]. Despite advances in systemic therapy and surgical techniques, the overall 5-year survival rate remains about 13% [2]. Surgical resection offers the only potentially curative treatment; however, only 10–20% of patients present with resectable and 20–25% with borderline resectable tumors, of whom approximately 70% become eligible for surgery after neoadjuvant chemotherapy [3, 4]. Even among those undergoing resection, nearly half relapse within the first postoperative year, effectively negating the curative intent and underscoring the need for reliable preoperative risk stratification [5–7].

Preoperative assessment in PDAC has evolved from subjective surgical judgment to standardized, imaging-based definitions of resectability. The 2006 National Comprehensive Cancer Network (NCCN) guideline introduced objective criteria for resectable, borderline resectable, and unresectable disease based on arterial and venous involvement on high-quality computed tomography (CT), later refined by international consensus statements adopting similar vessel contact thresholds (≤ or > 180°) and emphasizing vascular reconstructability [8–10]. The American Joint Committee on Cancer (AJCC) 8th edition (2017) subsequently refined pathological staging using tumor size-based T and node count-based N categories, improving prognostic precision but offering limited guidance for preoperative decisions [11]. Nevertheless, up to 20% of patients classified as resectable by NCCN criteria experience early recurrence within 6 months, resulting in futile surgery in roughly one-fifth of cases [12]. Moreover, real-world adherence to these frameworks remains suboptimal, with 50–70% of centers fully implementing the latest NCCN recommendations, particularly those relying on advanced imaging or genetic testing. In this context, treatment decisions between up-front surgery (US) and neoadjuvant therapy (NT) are frequently determined within a multidisciplinary tumor board and are guided by a combination of anatomic, biologic, and patient-related considerations, as no single guideline framework fully integrates all of these elements. In practice, these considerations may include institutional expertise, clinician judgment, patient performance status, and other clinical or imaging features suggestive of more aggressive tumor biology [13, 14].

Parallel efforts have explored biochemical and inflammatory markers to capture tumor aggressiveness. Serum CA19-9, although widely used, is variably accurate and confounded by cholestasis and Lewis antigen status [15–17]. Similarly, systemic inflammation indices such as the neutrophil-to-lymphocyte ratio (NLR), hypoalbuminemia, and C-reactive protein (CRP)-to-albumin ratio (CAR) reflect host–tumor interaction but demonstrate inconsistent predictive value [18]. A 2023 systematic review found that inflammatory scores alone were unreliable for predicting 12-month recurrence, suggesting that integration with tumor-specific anatomic metrics is essential for clinical applicability [19].

Recent advances in radiomics and machine-learning models combining imaging, serologic, and inflammatory variables offer promise in capturing tumor biology beyond anatomy, though widespread implementation remains limited [20]. We therefore hypothesized that combining easily obtained CT features with systemic inflammation markers would produce a practical bedside score that predicts 12-month recurrence better than existing anatomy- or biomarker-based systems.

## Methods

### Study design and setting

We conducted a retrospective cohort study at Firoozgar and Rasool-Akram Hospitals, two high-volume tertiary centers affiliated with Iran University of Medical Sciences (Tehran, Iran), and operating within the same university radiology network. Both centers share a unified perioperative pathway and a standardized pancreatic multidetector CT (MDCT) protocol, which was consistently applied throughout the study period. Medical records of all patients who underwent pancreaticoduodenectomy (Whipple procedure) between 1 April 2017 and 31 December 2024 were reviewed. The study was approved by the ethics committee of Iran University of Medical Sciences (IR.IUMS.REC.1402.813) and conducted in accordance with the Declaration of Helsinki. In accordance with national regulations, the requirement for informed consent was waived by the institutional ethics committee of Iran University of Medical Sciences because of the retrospective study design and the use of fully de-identified data.

### Patient population and eligibility

Eligible patients met the following inclusion criteria: (i) histopathologically confirmed PDAC on the resection specimen and (ii) availability of preoperative CT imaging for review. Exclusion criteria were: (i) irretrievable preoperative CT, (ii) unresectable disease at the time of imaging based on NCCN v2.2024 criteria, or (iii) a synchronous malignancy within the preceding five years. The analytic cohort was stratified into two pathways: up-front surgery (US) and post-neoadjuvant therapy (NT). Treatment pathway allocation had been determined by the same institutional multidisciplinary pancreatic tumor board, with stable membership and consistent operation across both centers throughout the study period. Throughout the 8-year study period, NCCN criteria remained the constant primary framework for assessing tumor resectability; however, final treatment decisions had not been restricted to a single predefined guideline.

Instead, decisions had integrated anatomical findings on CT with additional inflammatory and clinical indicators, as well as clinical judgment of both pancreatic surgeons and medical oncologists. Consequently, while patients with clearly resectable disease had generally undergone up-front surgery, neoadjuvant therapy had also been selectively administered in certain resectable cases when these multidisciplinary considerations suggested a potentially more aggressive disease course. Conversely, patients with borderline resectable features had been more likely to receive neoadjuvant therapy. Over time, emerging literature may have subtly influenced the board’s perspective, introducing minor temporal variation in pathway assignment that was not solely dictated by guideline‑based resectability. Patients who died within 30 days after surgery were excluded from all study analyses, as these early postoperative deaths were attributed to surgical complications rather than tumor biology.

### Preoperative MDCT acquisition and interpretation

All studies were acquired on 64- and 128-detector scanners (120 kVp; automated mA modulation), using an identical pancreatic CT acquisition protocol at both centers over the entire study period: non-enhanced axial (2 mm), late-arterial (~ 35 s), and portal-venous (~ 65 s) phases after intravenous injection of 100 mL non-ionic contrast (370 mg mL⁻¹ at 3–4 mL s⁻¹) followed by a 30 mL saline flush at the same rate. The arterial (pancreatic parenchymal) phase was triggered using automated bolus tracking with a region of interest placed in the abdominal aorta (threshold 100 HU). Images were reconstructed at 1.0–1.25 mm in axial and sagittal planes.

Two fellowship-trained abdominal radiologists (4 and 11 years of experience), blinded to outcomes and clinical data, independently reviewed the preoperative CT scans for resectability status according to NCCN v2.2024 criteria. Also, they completed a prespecified electronic case report form (eCRF); discrepancies were resolved by consensus. Continuous quantitative variables included maximal axial tumor diameter, perpendicular diameter, main pancreatic duct (MPD), common bile duct calibers, MPD-to-parenchymal thickness ratio (MPD/PT), and minimum arterial distance (smallest edge-to-edge separation across contiguous sections using a standardized tumor-margin definition). Qualitative variables included presence of necrosis (< 15 HU enhancement on arterial and portal phases), presence of peripancreatic fat stranding (blurred halos/linear attenuation adjacent to the tumor), presence of phase-specific hypoattenuation, enhancement pattern (homogeneous/heterogeneous/poor/rim), presence of adjacent-organ invasion, and circumferential vascular contact categorized in 90° increments (0–360°, coded 0–4) for portal vein (PV), superior mesenteric vein (SMV), SMV–PV confluence, superior mesenteric artery (SMA), celiac axis and major branches. Presence of nodal involvement was defined as either radiologically defined pathologic nodes based on size criteria (short-axis diameter > 6 mm at the porta hepatis or > 10 mm elsewhere) or CT-suspicious nodes showing round morphology, indistinct or irregular margins, central necrosis, or clustering of three or more nodes ≥ 5 mm within a single drainage basin. Both radiologically pathologic and suspicious lymph nodes were evaluated separately within the peripancreatic and “in-limit of resection” nodal basins (including the SMA, coeliac, and porta hepatis regions). To assess reproducibility of CT features, inter-observer agreement between the two radiologists was evaluated, by variable type, using Cohen’s κ coefficients for categorical variables or intraclass correlation coefficients (ICC; two-way random-effects, absolute-agreement, single-measure) for continuous measurements.

### Laboratory variables and inflammatory indices

Preoperative blood tests obtained within 28 days before surgery were retrieved for both cohorts; for NT group, the last post-therapy, pre-operative panel was used. Variables included complete blood count (CBC) with differential, platelet count, albumin, CRP, glucose, erythrocyte sedimentation rate (ESR), carcinoembryonic antigen (CEA), and CA19-9. Composite indices were calculated on their original scales as follows: NLR (neutrophil-to-lymphocyte ratio), CAR (CRP-to-albumin ratio), GAR (globulin-to-albumin ratio), and PAR (platelet-to-albumin ratio).

To minimize bias from inflammation related to biliary obstruction or the drainage procedure, values obtained before biliary drainage or < 14 days post-drainage were excluded for sensitive variables (CBC with differential, platelet count, ESR, CRP). For patients without drainage, we used the most recent preoperative labs measured ≤ 28 days before surgery. For patients with drainage, we used the most recent value that was both > 14 days post-drainage and ≤ 28 days before surgery. Otherwise, variables were marked missing.

### Variables, outcomes, and data handling

Continuous variables, including biochemical indices and quantitative CT parameters, were analyzed on their original scale without applying arbitrary cut-off points. Potential non-linear associations with recurrence risk were examined using restricted cubic splines. Binary variables, including the presence or absence of CT features, were coded as 0/1. Ordinal vascular contact was coded on a 0–4 scale corresponding to 90° increments (0–360°), and the enhancement pattern was treated as a four-level categorical variable (homogeneous, heterogeneous, poor, or rim).

The primary outcome was short-term recurrence-free survival, defined as the time from resection to the first radiologic or histologic evidence of locoregional or distant recurrence within 12 months after surgery. Death without prior recurrence was considered non-informative censoring, and patients alive and recurrence-free were censored at the last follow-up (31 December 2024).

The secondary outcome was 12-month recurrence status, treated as a binary endpoint. Although prior studies have used variable thresholds (commonly 6–12 months) and no uniform definition exists, we selected 12 months a priori for the short-term analysis based on literature indicating this timepoint optimally discriminates subsequent prognosis [21].

Missing data were addressed using multiple imputation by chained equations with 20 datasets and 10 iterations, performed after temporal data-splitting and separately within each analysis pathway (US and NT) to avoid information leakage; treatment pathway was therefore not included as an imputation predictor. Within-pathway imputation models included all covariates and the outcome indicator to ensure congeniality. Predictive mean matching was used for continuous variables, logistic regression for binary variables, and proportional-odds models for ordered factors. Estimates from the imputed datasets were pooled using Rubin’s rules.

Convergence diagnostics were evaluated to confirm stability and plausibility, and complete-case analyses were performed as sensitivity checks.

### Statistical analysis and modeling

All analyses were performed in R 4.3.2 (survival, rms, mice, glmnet, and timeROC packages), with fixed random seeds. All analyses were conducted separately for the US and NT pathways.

Baseline characteristics were summarized as either mean ± standard deviation (SD) or median ± interquartile range (IQR) and compared with Welch t-, Mann–Whitney U-, or χ² tests. Data were divided chronologically into a 70% development set (patients treated before October 2022 for the NT cohort and before December 2022 for the US cohort) and a 30% temporal test set (patients treated on or after those dates, respectively). Candidate predictors included quantitative and qualitative CT descriptors and preoperative biochemical and inflammatory indices, modeled as described in Sect.  2.3–2.5. Missing data were handled by multiple imputation with chained equations (20 datasets, 10 iterations), performed separately within each pathway after the temporal split. For each imputed dataset, univariable Cox (for recurrence-free survival) and logistic (for 12-month recurrence) regressions were performed; predictors with *P* < 0.10 or clinical relevance were entered into multivariable modeling.

Primary predictive models were pathway-specific multivariable logistic regressions targeting 12-month recurrence. Backward selection guided by Akaike’s information criterion identified the final variable set, followed by ridge penalization (λ via 10-fold cross-validation) to stabilize coefficients. Internal validation used 1,000 bootstrap resamples to estimate optimism-corrected discrimination and calibration. Model performance was assessed in the temporal test subset by the area under the receiver operating characteristic curve (AUC), calibration intercept and slope, and Brier score, with comparative performance versus AJCC 8th edition evaluated using DeLong tests, continuous net reclassification improvement (cNRI), and integrated discrimination improvement (IDI). Coefficients from the final ridge-stabilized models were scaled and rounded to generate pathway-specific point scores [22], stratified into three prespecified risk groups reflecting approximate 1-year recurrence probabilities. Clinical utility was quantified by decision-curve analysis across threshold probabilities of 0.15–0.60. Sensitivity analyses included complete-case modeling, alternative endpoints (9-month recurrence), and predictor-set ablation to confirm robustness.

To address the potential for variable-selection instability inherent to multi-step modelling in small development cohorts, we conducted a dedicated bootstrap stability analysis on the development sets (US, *n* = 57; NT, *n* = 36). The complete variable-selection pipeline - univariable screening at *P* < 0.10, AIC-guided backward elimination, and ridge penalisation - was re-applied to each of 1,000 bootstrap resamples drawn with replacement from the respective development cohort, and the proportion of valid resamples (≥ 4 events and ≥ 4 non-events) in which each candidate predictor survived to the final model was recorded as its bootstrap selection frequency. A threshold of ≥ 50% was pre-specified as the empirical criterion for selection stability. Bootstrap distributions of unpenalised regression coefficients for the final-model predictors were additionally examined to assess consistency of effect direction and magnitude; the proportion of resamples in which a predictor’s coefficient reversed sign relative to the full-sample estimate was recorded as an index of directional reliability.

## Results

### Cohort assembly

**Fig. 1:**
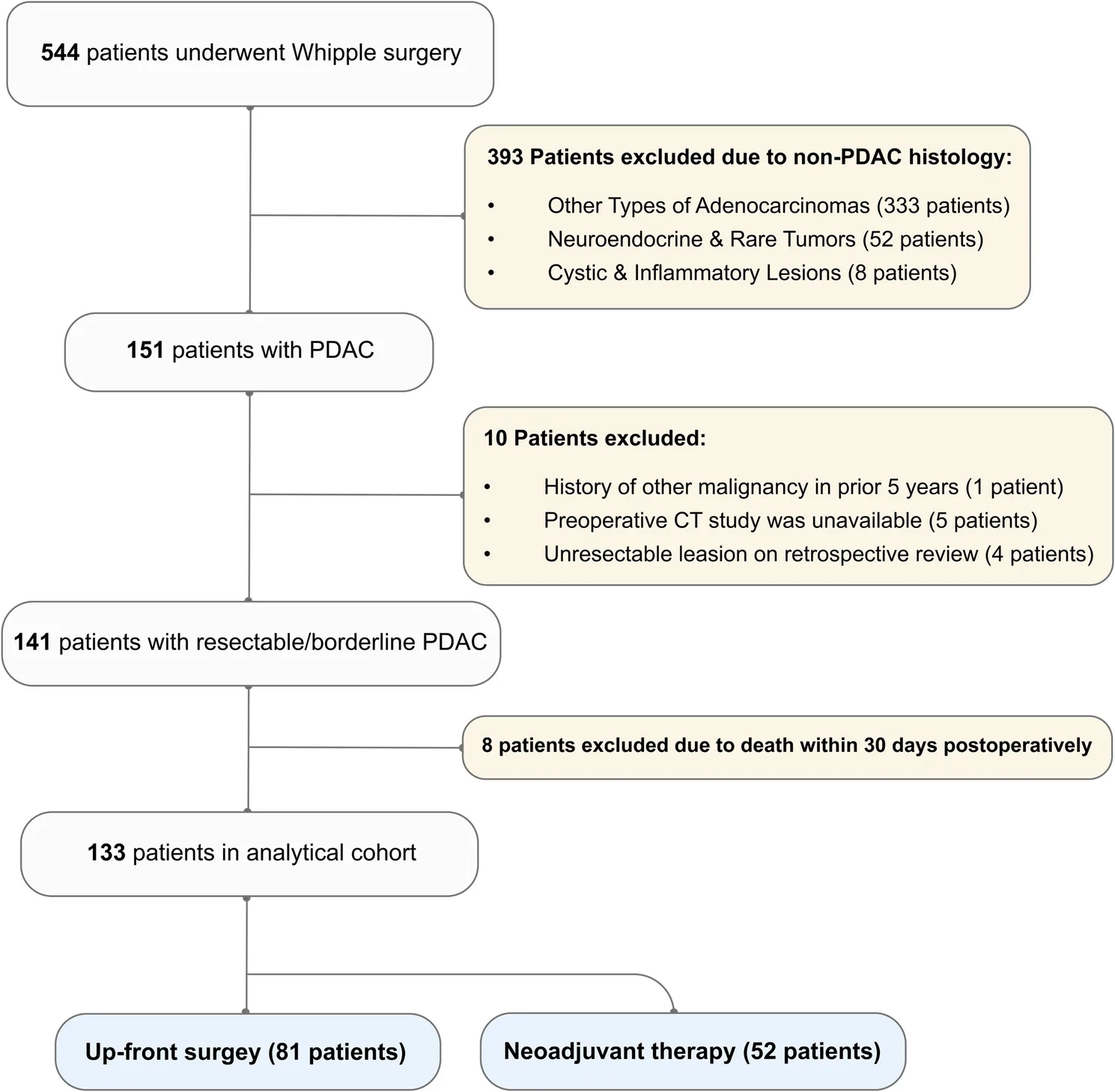
Study flow diagram. Cohort assembly from initial screening (2017–2024) through exclusions to the final analysis set, with stratification by treatment pathway (up-front surgery [US], neoadjuvant therapy [NT])

As shown in Fig. 1, among 544 patients who underwent pancreaticoduodenectomy between 2017 and 2024, 133 resected cases with histologically confirmed PDAC met eligibility criteria (US, *n* = 81; NT, *n* = 52). A stratified 70/30 temporal split produced development cohorts (US, *n* = 57; NT, *n* = 36) and test cohorts (US, *n* = 24; NT, *n* = 16). The mean age was 65.4 years in the US group and 60.1 years in the NT group, with female representation of 44.4% and 34.6%, respectively. Baseline demographic variables did not differ significantly between patients with and without recurrence (Supplementary File, Table S1). As detailed in Supplementary Table S2, item-level missingness was modest (median 3.8%, IQR 0.0–7.1%; maximum 11.3% for CRP), with imaging variables missing in 0–4.5% and laboratory indices in 0–11.3% of cases. Overall, 27% of patients had at least one missing predictor. Multiple imputation by chained equations was applied within each pathway after the temporal split to preserve power and minimize selection bias, incorporating all covariates and the outcome indicator. Convergence diagnostics, including iteration trace plots and fraction of missing information values below 0.3 for all variables, confirmed model stability and satisfactory convergence of the imputation process.

### Excluded cases

As detailed in Supplementary Figure S1, 393 patients were excluded due to non-PDAC histology, including neoplastic entities (most commonly intestinal type adenocarcinoma and distal cholangiocarcinoma) and non-neoplastic conditions, primarily chronic pancreatitis. These patients had initially presented with clinical and radiological findings suggestive of a periampullary malignancy—most commonly a pancreatic head or periampullary mass associated with biliary and/or pancreatic ductal dilatation—and were therefore considered candidates for pancreaticoduodenectomy. In a few atypical cases, the final diagnoses included gallbladder carcinoma involving the left sub-hepatic gallbladder neck, porta hepatis, deudenum, and periampullary region; pancreatic lymphoma presenting as an ill-defined hypo enhancing pancreatic head lesion with enlarged peripancreatic lymph nodes but without biliary or pancreatic duct obstruction; gastric adenocarcinoma with direct extension from the antrum to the pancreatic head; and a large necrotic medial deudenal wall gastrointestinal stromal tumor (GIST) with pancreatic involvement.

Among patients with PDAC histology, one was excluded due to synchronous malignancy within five years (breast cancer metastatic to the lung). Nine additional patients were excluded based on preoperative CT criteria: five due to unavailable CT studies for retrospective review, and four due to findings consistent with unresectable disease on retrospective reassessment using NCCN v2.2024 criteria, despite initial surgical exploration. Three of these patients had unrecognized 180–270° tumor abutment of the common hepatic artery and underwent pancreatoduodenectomy with arterial and venous reconstruction, while the fourth patient had > 180° tumor abutment of the SMA trunk, underwent pancreatoduodenectomy with periarterial SMA dissection, but intraoperative findings revealed liver involvement requiring segmentectomy. Resectability was determined by consensus of two radiologists, whose independent assessments showed excellent inter-observer agreement (Cohen’s κ = 0.88). Finally, eight patients with resectable PDAC on preoperative CT were excluded due to early postoperative death (within 30 days), caused by operation related complications including, sepsis (*n* = 4), massive cerebrovascular accident (*n* = 2), disseminated intravascular coagulation with severe hemorrhage (*n* = 1), and massive pulmonary embolism with cardiac arrest (*n* = 1). Detailed characteristics of these patients are reported in Supplementary Table S3.

### Associations of CT and inflammatory biomarkers with early recurrence

As in Table 1, across both treatment pathways, multiple CT features served as surrogates of aggressive tumor biology associated with early relapse. In the US cohort, larger tumor size (per mm HR 1.08, 95% CI 1.04–1.13) and portal-venous hypodensity were significant adverse markers. Intratumoral necrosis (HR 3.63, 95% CI 1.95–6.77) and peripancreatic fat stranding (HR 3.42, 95% CI 1.91–6.15) exhibited the strongest associations with recurrence, while heterogeneous, poor, or rim enhancement patterns further increased risk. In the NT group, tumor size (per mm HR 1.17, 95% CI 1.10–1.25), delayed-phase hypodensity (HR 3.74, 95% CI 1.35–10.33), necrosis (HR 3.96, 95% CI 1.72–9.11), and rim enhancement (HR 5.90, 95% CI 2.10–16.56) were the most prominent predictors. Inter‑observer agreement for CT features was substantial to almost perfect (κ = 0.75–0.82; ICC = 0.77–0.85).

**Table 1:** Univariable CT morphology and attenuation, by pathway

Variable	Up-front surgery					Neoadjuvant therapy				
	With recurrence (*n* = 47)	Without recurrence (*n* = 34)	Between groups *P*	HR (95% CI)	Univariable *P*	With recurrence (*n* = 23)	Without recurrence (*n* = 29)	Between groups *P*	HR (95% CI)	Univariable *P*
Maximum diameter, mm (mean ± SD)	35.0 ± 6.9	29.1 ± 7.6	< 0.01	1.08 (1.04–1.13)	< 0.01	36.4 ± 6.3	28.4 ± 4.1	< 0.01	1.17 (1.10–1.25)	< 0.01
Diameter ≥ 30 mm, n (%)	34 (72.3)	15 (48.4)	0.054	2.10 (1.11–3.99)	0.02	20 (90.9)	13 (46.4)	< 0.01	6.77 (1.58–29.04)	0.01
Tumor area, mm² (mean ± SD)	994 ± 390	711 ± 433	< 0.01	1.001 (1.000–1.002)	< 0.01	1029 ± 362	675 ± 222	< 0.01	1.003 (1.001–1.004)	< 0.01
Dominant location, n (%)			0.15					0.29		
Head	23 (48.9)	17 (50.0)		Reference		5 (21.7)	11 (37.9)		Reference	
Head + neck	5 (10.6)	0		1.45 (0.55–3.85)	0.46	0	1 (3.4)		10.88 (1.15–102.54)	0.04
Head + uncinate	18 (38.3)	17 (50.0)		0.89 (0.48–1.64)	0.70	14 (60.9)	17 (58.6)		1.78 (0.64–4.95)	0.27
Body–tail	1 (2.1)	0		2.21 (0.30–16.54)	0.44	3 (13.0)	1 (3.4)		2.89 (0.69–12.11)	0.15
Tumor conspicuity, n (%)			0.22					0.02		
Well / moderate	11 (23.4)	13 (38.2)		Reference		5 (21.7)	16 (55.2)		Reference	
Poor	36 (76.6)	21 (61.8)		1.63 (0.83–3.21)	0.16	18 (78.3)	13 (44.8)		3.27 (1.21–8.84)	0.02
Peripancreatic fat stranding, n (%)	26 (55.3)	4 (11.8)	< 0.01	3.42 (1.91–6.15)	< 0.01	15 (65.2)	4 (13.8)	< 0.01	6.97 (2.89–16.78)	< 0.01
Attenuation vs. parenchyma — unenhanced, n (%)			0.12					1		
Iso-attenuating	32 (68.1)	29 (85.3)		Reference		20 (87.0)	25 (86.2)		Reference	
Hypodense	15 (31.9)	5 (14.7)		1.49 (0.81–2.75)	0.20	3 (13.0)	4 (13.8)		1.03 (0.31–3.47)	0.96
Attenuation — late arterial phase, n (%)			0.16					0.64		
Iso- attenuating	3 (6.4)	6 (17.6)		Reference		3 (13.0)	2 (6.9)		Reference	
Hypodense	44 (93.6)	28 (82.4)		1.83 (0.57–5.90)	0.31	20 (87.0)	27 (93.1)		0.57 (0.17–1.91)	0.36
Attenuation portal-venous phase, n (%)			< 0.01					0.15		
Iso-attenuating	5 (10.6)	15 (44.1)		Reference		5 (21.7)	12 (41.4)		Reference	
Hypodense	42 (89.4)	19 (55.9)		3.45 (1.36–8.73)	< 0.01	18 (78.3)	17 (58.6)		2.19 (0.81–5.91)	0.12
Attenuation — delayed phase, n (%)			0.12					0.22		
Iso-attenuating	26 (55.3)	25 (73.5)		Reference		18 (78.3)	27 (93.1)		Reference	
Hypodense	21 (44.7)	9 (26.5)		1.44 (0.81–2.56)	0.21	5 (21.7)	2 (6.9)		3.74 (1.35–10.33)	0.01
ΔHU > 15 (late arterial), n (%)	39 (83.0)	13 (38.2)	< 0.01	3.67 (1.71–7.90)	< 0.01	17 (73.9)	21 (72.4)	1	1.01 (0.40–2.56)	0.99
Tumor necrosis present, n (%)	32 (68.1)	7 (20.6)	< 0.01	3.63 (1.95–6.77)	< 0.01	11 (47.8)	4 (13.8)	< 0.01	3.96 (1.72–9.11)	< 0.01
Enhancement pattern, n (%)			< 0.01					0.07		
Homogeneous	9 (19.1)	22 (64.7)		Reference		10 (43.5)	18 (62.1)		Reference	
Heterogeneous	14 (29.8)	5 (14.7)		3.23 (1.39–7.52)	< 0.01	4 (17.4)	5 (17.2)		1.62 (0.51–5.18)	0.41
Poor	10 (21.3)	2 (5.9)		5.11 (2.05–12.77)	< 0.01	4 (17.4)	6 (20.7)		1.18 (0.37–3.75)	0.78
Rim	14 (29.8)	5 (14.7)		3.27 (1.41–7.58)	< 0.01	5 (21.7)	0		6.72 (2.20–20.55)	< 0.01
Rim enhancement (late arterial), n (%)	14 (29.8)	5 (14.7)	0.18	1.51 (0.81–2.81)	0.20	5 (21.7)	0	0.01	5.90 (2.10–16.56)	< 0.01
Adjacent-organ invasion present, n (%)	34 (72.3)	15 (44.1)	0.01	2.22 (1.17–4.23)	0.01	15 (65.2)	19 (65.5)	1	0.91 (0.39–2.14)	0.83

Univariable associations between pre-operative CT features and recurrence. Effect measures are hazard ratios (HR) for recurrence-free survival with 95% CIs from Cox models

As in Table 2, systemic inflammatory and serologic indices provided complementary prognostic information. In the US group, higher neutrophil-to-lymphocyte ratio (NLR; HR 2.05, 95% CI 1.20–3.50) and CRP-to-albumin ratio (CAR; HR 4.48, 95% CI 2.08–9.65) independently correlated with recurrence. In the NT group, low albumin was protective (HR 0.54, 95% CI 0.30–0.98), while elevated CEA (HR 2.26, 95% CI 1.18–4.22), NLR (HR 1.87, 95% CI 1.02–3.42), CAR (HR 6.42, 95% CI 1.81–22.7), and GAR (HR 1.98, 95% CI 1.01–3.87) were adverse prognostic factors.

**Table 2:** Systemic-inflammatory and serologic markers, by pathway

Variable	Up-front surgery					Neoadjuvant therapy				
	With recurrence (*n* = 47)	Without recurrence (*n* = 34)	Between groups *P*	HR (95% CI)	Univariable *P*	With recurrence (*n* = 23)	Without recurrence (*n* = 29)	Between groups *P*	HR (95% CI)	Univariable *P*
WBC count (×10⁹ L⁻¹)	7.45 ± 2.51	7.01 ± 1.73	0.35	1.22 (0.78–1.89)	0.38	8.29 ± 2.77	7.09 ± 1.73	0.08	1.73 (0.96–3.14)	0.07
Neutrophil count (%)	70.78 ± 12.06	60.08 ± 12.17	< 0.01	2.58 (1.48–4.49)	< 0.01	62.57 ± 19.29	62.43 ± 10.36	0.98	1.01 (0.59–1.74)	0.97
Lymphocyte count (%)	21.34 ± 10.37	30.40 ± 9.95	< 0.01	0.41 (0.24–0.68)	< 0.01	23.15 ± 10.48	26.82 ± 8.38	0.18	0.67 (0.38–1.19)	0.17
Platelet count (×10⁹ L⁻¹)	257.70 ± 92.75	258.35 ± 76.23	0.97	0.99 (0.64–1.53)	0.97	250.7 ± 77.7	214.1 ± 61.6	0.07	1.74 (0.95–3.20)	0.07
Albumin (g dL⁻¹)	3.43 ± 0.58	3.66 ± 0.56	0.07	0.67 (0.42–1.05)	0.08	3.58 ± 0.55	3.89 ± 0.48	0.04	0.54 (0.30–0.98)	0.04
Glucose (mg dL⁻¹)	128.3 ± 49.1	120.4 ± 44.7	0.45	1.18 (0.72–1.90)	0.51	141.0 ± 52.6	128.5 ± 44.3	0.30	1.30 (0.70–2.42)	0.40
ESR (mm h⁻¹)	28.6 ± 16.7	25.0 ± 14.8	0.32	1.14 (0.70–1.84)	0.60	32.4 ± 18.1	28.5 ± 16.8	0.42	1.50 (0.69–3.26)	0.30
CRP (mg L⁻¹)	22.0 ± 28.8	7.4 ± 10.9	< 0.01	1.46 (0.88–2.41)	0.14	16.0 ± 20.3	9.4 ± 15.1	0.15	1.61 (0.79–3.28)	0.19
CA19-9 (U mL⁻¹)	565 ± 822	365 ± 719	0.32	1.31 (0.79–2.17)	0.30	769 ± 1 080	509 ± 809	0.13	1.83 (0.94–3.53)	0.07
CEA (ng mL⁻¹)	8.1 ± 11.2	5.0 ± 6.8	0.19	1.37 (0.82–2.31)	0.23	8.9 ± 10.6	4.3 ± 3.1	0.01	2.26 (1.18–4.22)	0.02
NLR	3.60 ± 1.23	2.29 ± 0.96	< 0.01	2.05 (1.20–3.50)	< 0.01	3.31 ± 1.33	2.29 ± 1.00	0.01	1.87 (1.02–3.42)	0.04
PAR	8.52 ± 3.71	7.60 ± 3.23	0.21	0.87 (0.54–1.41)	0.58	9.28 ± 4.43	7.40 ± 3.14	0.09	1.35 (0.75–2.45)	0.32
GAR	39.9 ± 26.1	34.4 ± 24.1	0.36	0.84 (0.53–1.32)	0.44	54.6 ± 27.1	39.1 ± 19.0	0.03	1.98 (1.01–3.87)	0.04
CAR	11.24 ± 10.40	2.24 ± 3.64	< 0.01	4.48 (2.08–9.65)	< 0.01	7.23 ± 8.85	1.98 ± 2.03	0.01	6.42 (1.81–22.7)	0.01

Univariable associations for pre-operative laboratory markers and composite indices, including albumin, Continuous variables analyzed on native scales unless otherwise specified

*CRP *C-Reactive Protein, *CEA* Carcinoembryonic antigen,* ESR *Erythrocyte sedimentation rate, CA19-9, and derived metrics,* NLR *Neutrophil-to-lymphocyte ratio, *CAR *C-reactive protein-to-albumin ratio* , GAR *Globulin-to-albumin ratio *, PAR *Platelet-to-albumin ratio

HRs (95% CIs) for recurrence-free survival are reported

### Multivariable models and bedside point-score construction

Pathway-specific multivariable logistic models were developed to predict 12-month recurrence, integrating complementary radiologic and inflammatory predictors (Table 3). In the US group, tumor size, intratumoral necrosis, peripancreatic fat stranding, and elevated CAR and NLR remained independent predictors after penalization. In the NT group, tumor size, hepatic-artery contact ≥ 90°, and higher CAR, NLR, and CEA levels were retained.

**Table 3:** Final multivariable models and bedside point allocation

Predictor	Up-front surgery					Neoadjuvant therapy				
	β	OR (95% CI)	Wald *P*	Selection (*n*/20)	Points	β	OR (95% CI)	Wald *P*	Selection (*n*/20)	Points
Diameter (per 5 mm)	0.23	1.26 (1.05–1.52)	0.01	20	+ 1 per 5 mm > 25 (max 4)	0.28	1.33 (1.05–1.70)	0.02	20	+ 1 per 5 mm > 25 (max 4)
Tumor necrosis (yes)	1.05	2.86 (1.33–6.14)	< 0.01	19	4	—	—	—	—	—
Peripancreatic fat stranding (yes)	1.24	3.46 (1.52–7.90)	< 0.01	18	5	—	—	—	—	—
Hepatic-artery contact ≥ 90°	—	—	—	—	—	1.31	3.71 (1.27–10.9)	0.02	18	4
log₂-CAR	0.57	1.77 (1.17–2.69)	< 0.01	17	+ 2 (≥ 2)	0.9	2.46 (1.30–4.66)	< 0.01	17	+ 3 (≥ 2)
log₂-NLR	0.49	1.63 (1.02–2.59)	0.04	12	+ 2 (≥ 1.6)	0.55	1.73 (1.01–2.97)	0.04	11	+ 2 (≥ 1.6)
log₂-CEA	—	—	—	—	—	0.62	1.86 (1.04–3.32)	0.03	13	+ 2 (≥ 2.3)

Logistic models for 12-month recurrence, developed separately for up-front surgery (US) and neoadjuvant-therapy (NT) pathways

Candidate selection used AIC-guided backward elimination; final coefficients estimated under a fixed ridge penalty (λ via 10-fold CV) to stabilize estimates without changing variable membership. Intercepts and optimism-corrected C-statistics and calibration slopes are shown as reported in the original tables. The rightmost block provides the integer point allocation, obtained by dividing each non-zero β by the smallest non-zero β and rounding; summed points yield the bedside scores used throughout

Displayed for each pathway: adjusted coefficients (β), standard errors, Wald *P* values, and odds ratios (95% CI).

Coefficients were stabilized with ridge penalization to improve model robustness. For each pathway, each β coefficient was divided by the smallest non-zero β in that pathway (Diameter for both pathways), and the result rounded to the nearest integer to give each variable a point weight; point weights were summed to yield a total score reflecting the preoperative probability of 12-month recurrence (Table 3). These models served as the foundation for downstream validation and performance benchmarking analyses. Table 4 reports the number of recurrence events within each risk stratum and the events‑per‑variable for the final model in each pathway; in the development set, the US model had 18 recurrences and 5 predictors (events‑per‑variable = 3.6), and the NT model had 11 recurrences and 5 predictors (events‑per‑variable = 2.2), both below the commonly recommended minimum of 10 events per variable.

**Table 4:** The number of recurrence events in development sets in each risk stratum and the events‑per‑variable for the final model in each pathway

Study group	Patients	Recurrence events, *n* (%)	No. of variables in final model	Event-per-variable ratio
Up-front surgery	57	18 (31.6%)	5	3.6
Favorable	25	1 (4.0%)	-	-
Intermediate	20	8 (40.0%)	-	-
Poor	12	9 (75.0%)	-	-
Neoadjuvant therapy	36	11 (30.6%)	5	2.2
Favorable	15	2 (13.3%)	-	-
Intermediate	14	4 (28.6%)	-	-
Poor	7	5 (71.4%)	-	-

### Apparent and optimism-corrected performance in development

In the development cohorts, model calibration closely followed the ideal 45° reference line, with minimal optimism after bootstrap correction (Supplementary File, Figure S2). Discriminative performance was high and consistent with optimism-adjusted estimates, yielding AUC values of 0.84 (95% CI, 0.75–0.92) for the US model and 0.81 (95% CI, 0.68–0.92) for the NT model (Supplementary File, Figure S3). Calibration slopes were near unity (US, 0.94; NT, 0.92), and Brier scores were low (0.17–0.19), indicating strong discrimination and accurate absolute risk estimation (Supplementary File, Table S4).

### Bootstrap variable-selection stability

**Fig. 2:**
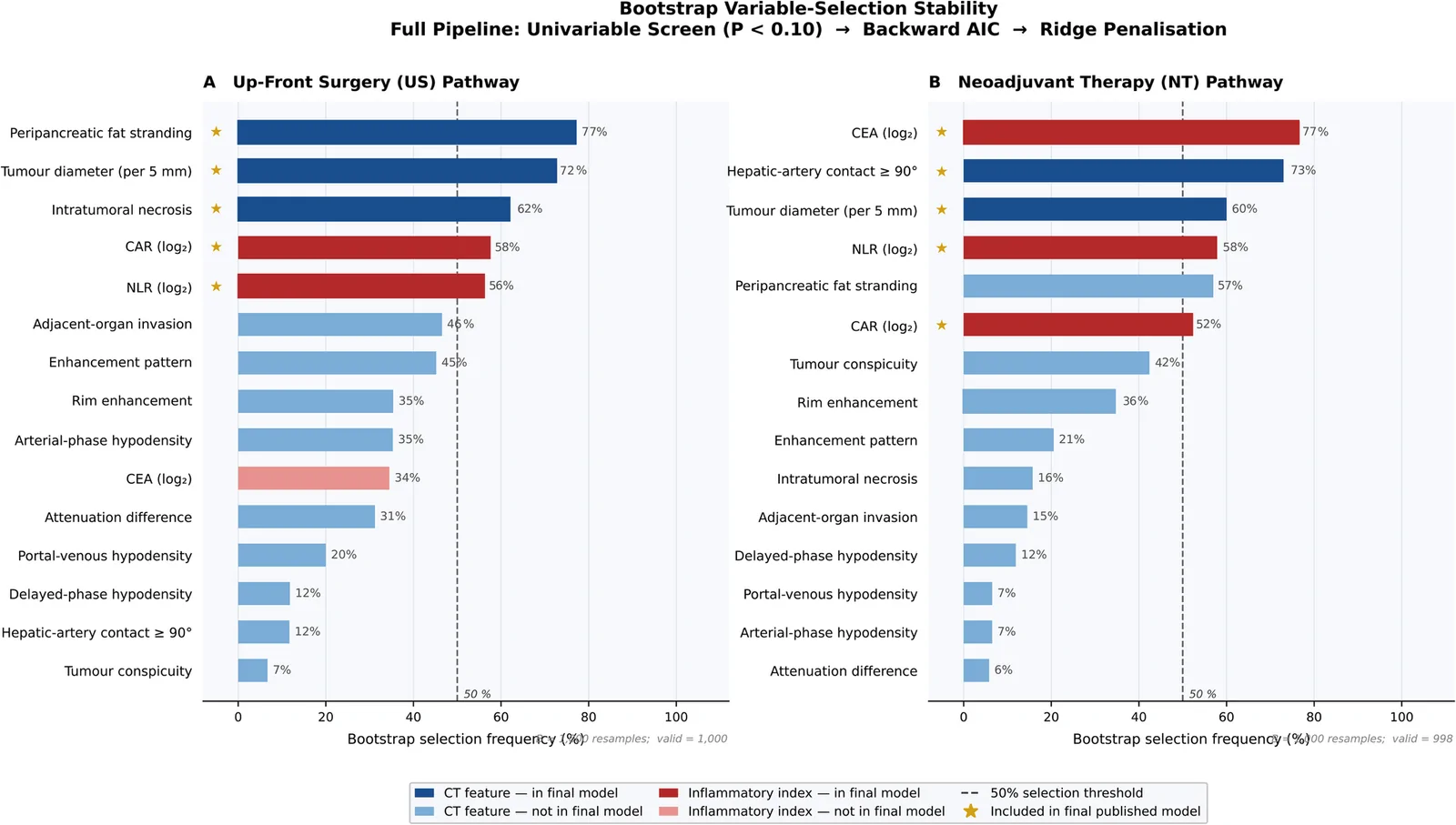
Bootstrap variable-selection stability. Selection frequency (%) of each candidate predictor after reapplying the full pipeline (univariable *P* < 0.10 → backward AIC → ridge) to 1000 bootstrap resamples, for (**A**) up-front surgery (US) and (**B**) neoadjuvant therapy (NT) pathways. Dashed line = 50% stability threshold. Highlighted symbols indicate predictors retained in the final published model

Bootstrap variable-selection frequencies across 1,000 resamples are shown in Fig. 2. In the US pathway, all predictive factors of the final model (including the three CT structural features and two inflammatory indices) exceeded the pre-specified 50% stability threshold. No non-final candidate met the threshold, with adjacent-organ invasion achieving the highest frequency among excluded variables (46.2%). Similarly, in the NT pathway, all predictors of the final model exceeded 50% stability threshold. Peripancreatic fat stranding was the most frequently selected non-final NT candidate (56.9%), likely reflecting pathway-specific rather than universal prognostic relevance.

Bootstrap distributions of unpenalized regression coefficients are shown in Supplementary Figure S4. CT structural features demonstrated consistent directionality in both pathways, with sign-change rates well below 15% throughout. Inflammatory indices showed greater instability: in the US pathway, log₂-NLR reversed sign in 34% of resamples and log₂-CAR in 23%; in the NT pathway, rates were 37% and 29%, respectively. Tumor diameter in the NT pathway showed a sign-change rate of 29%, likely a consequence of sparse events and bootstrap samples in which recurrences clustered disproportionately among smaller tumors.

### Clinical stratification and time-to-event separation

Risk strata were predefined from the pathway-specific bedside scores to represent low (favorable), intermediate, and high (poor) recurrence risk categories. Cut-points were determined a priori to approximate ≈ 70% and ≈ 20% predicted 1-year recurrence-free survival and were not tuned to observed outcomes. In the US group, score ranges of 0–4, 5–9, and 10–15 points defined the three strata, while in the NT group, strata were defined as 0–3, 4–8, and 9–15 points.

**Fig. 3:**
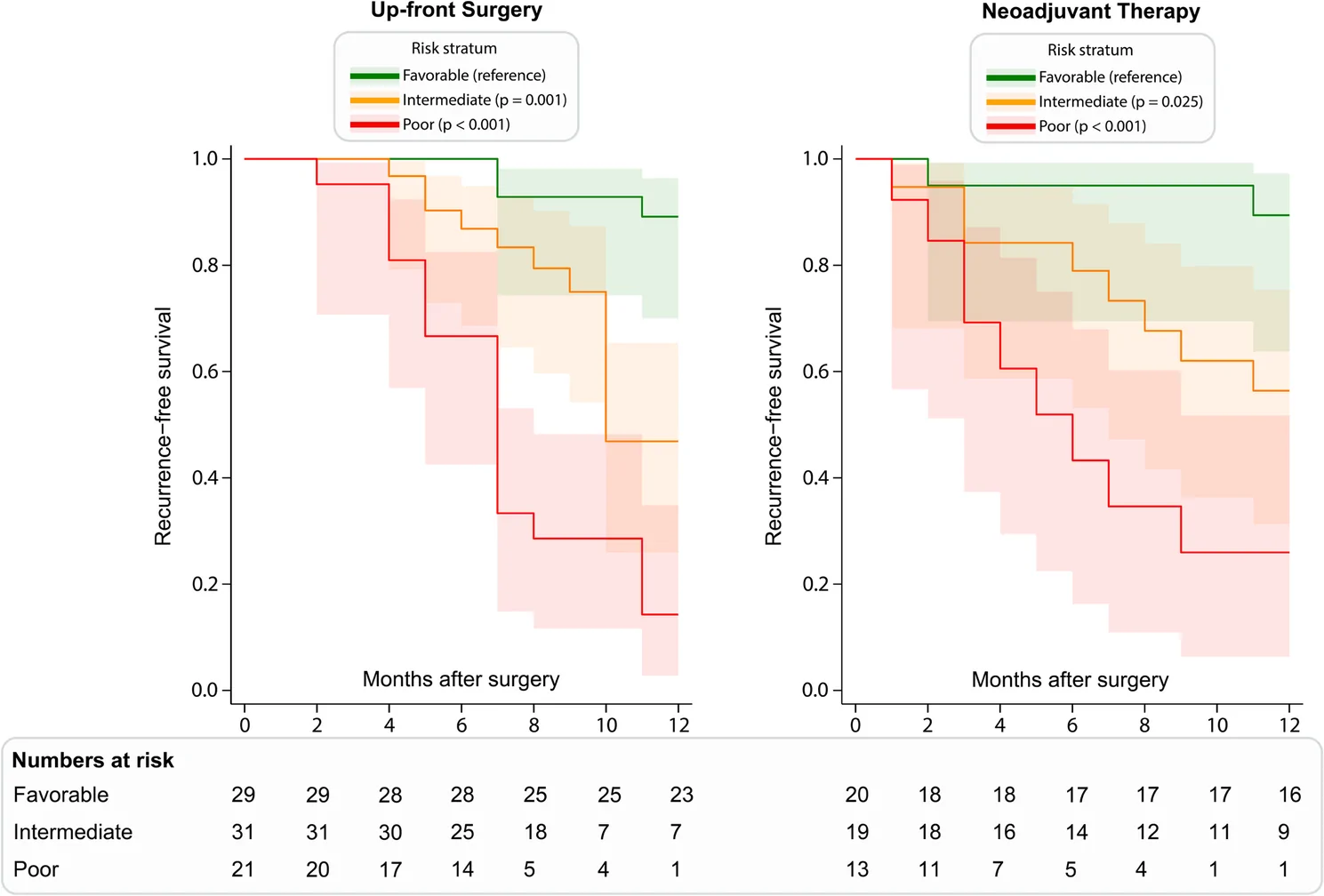
Kaplan–Meier recurrence-free survival by risk strata. Meier curves for 12‑month recurrence‑free survival stratified by risk score (Favorable, Intermediate, Poor) in the Up‑front Surgery (US) and Neoadjuvant Therapy (NT) cohorts (left and right panels, respectively). Solid lines are Kaplan–Meier estimates and shaded bands show pointwise 95% confidence intervals (calculated by Greenwood’s estimator); numbers at risk are reported below each panel. P values shown in the panel legends are from log‑rank comparisons versus the favorable (reference) stratum. patients without the recurrence (including death prior to recurrence) were censored at date of last known contact or at study end

**Fig. 4:**
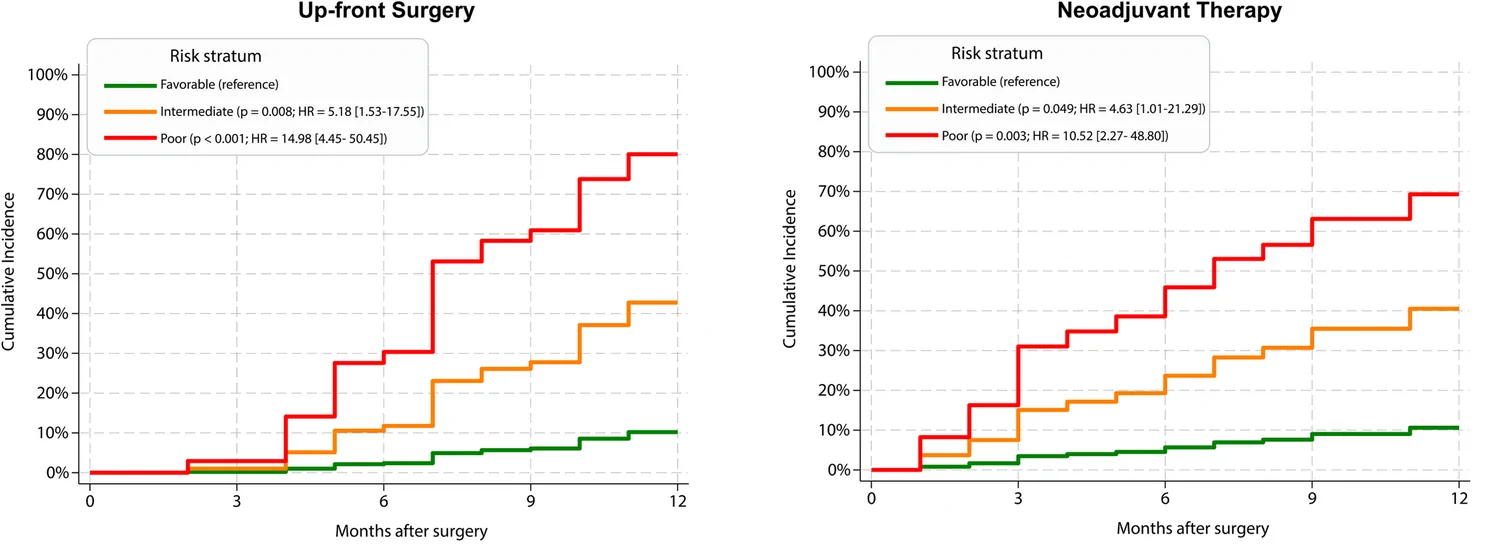
Cumulative incidence of recurrence within 12 months after surgery, stratified by preoperative risk score calculated by Fine-Gray model. Curves show estimated cumulative incidence functions; panel legends report p values for comparisons versus the favorable (reference) stratum and subdistribution hazard ratios from Fine–Gray competing-risks models (reference = favorable): Up-front Surgery — Intermediate sHR 5.18 (95% CI 1.53–17.55; *p* = 0.008) and Poor sHR 14.98 (95% CI 4.45–50.45; *p* < 0.001); Neoadjuvant Therapy — Intermediate sHR 4.63 (95% CI 1.01–21.29; *p* = 0.049) and Poor sHR 10.52 (95% CI 2.27–48.80; *p* = 0.003). Deaths without documented recurrence were treated as competing events; patients without recurrence or competing events were censored at last follow-up

Across both pathways, recurrence rates increased markedly with higher strata (Table 5). In US, 12-month recurrence rose from 10.3% in the favorable group to 41.9% and 81.0% in the intermediate and poor-risk groups, respectively (OR 5.9, 95% CI 1.4–24.2 and OR 29.3, 95% CI 6.4–134.2 vs. favorable). In NT, corresponding rates were 10.0%, 36.8%, and 69.2% (OR 4.9, 95% CI 0.9–25.5 and OR 18.7, 95% CI 3.0–116.4 vs. favorable). Kaplan–Meier curves showed clear temporal separation between strata in both pathways (Fig. 3), and predicted-risk distributions aligned monotonically with increasing score categories (Supplementary File, Figure S5), confirming strong discrimination and calibration of the preoperative scoring models. In Cox proportional‑hazards analysis, compared with the favorable group, the US cohort showed HRs of 5.90 (95% CI 1.67–20.90; *p* = 0.006) for the intermediate stratum and 15.71 (95% CI 4.53–54.42; *p* < 0.001) for the poor stratum, while the NT cohort showed HRs of 4.87 (95% CI 1.03–22.98; *p* = 0.045) and 13.08 (95% CI 2.74–62.38; *p* = 0.001), respectively. Because Kaplan–Meier treats deaths without prior recurrence as non-informative censoring, it can bias recurrence estimates when such competing events occur; we therefore performed a Fine–Gray competing-risks analysis, which models cumulative incidence while accounting for death as a competing event, and obtained similar results (Fig. 4). In the Fine–Gray competing‑risks analysis, the US cohort showed subdistribution hazard ratios (sHR) of 5.18 (95% CI 1.53–17.55; *p* = 0.008) for the intermediate stratum and 14.98 (95% CI 4.45–50.45; *p* < 0.001) for the poor stratum, while the NT cohort showed sHRs of 4.63 (95% CI 1.01–21.29; *p* = 0.049) and 10.52 (95% CI 2.27–48.80; *p* = 0.003), respectively. These results closely parallel the primary logistic and Cox findings, confirming that the risk stratification remains robust when accounting for death as a competing event.

**Table 5:** Twelve-month recurrence by bedside risk strata

Cohort	Score stratum	Points	Patients, *n*	Recurrences, *n* (%)	OR (95% CI)	*P*
Up-front Surgery	Favorable	0–4	29	3 (10.3)	Reference	–
	Intermediate	5–9	31	13 (41.9)	5.9 (1.4–24.2)	0.01
	Poor	10–15	21	17 (81.0)	29.3 (6.4–134.2)	< 0.01
Neoadjuvant therapy	Favorable	0–3	20	2 (10.0)	Reference	–
	Intermediate	4–8	19	7 (36.8)	4.9 (0.9–25.5)	0.07
	Poor	9–15	13	9 (69.2)	18.7 (3.0–116.4)	< 0.01

Outcomes according to three pre-specified bedside score strata (favorable / intermediate / poor) in each pathway. Shown are group sizes (n, %), observed 12-month recurrence with 95% CIs, and ORs versus the favorable stratum

### Generalization to temporal test sets and benchmark versus AJCC

In the held-out test cohorts, bedside scores demonstrated superior discrimination compared with AJCC staging. In the US group, the AUC was 0.82 (95% CI, 0.66–0.95) versus 0.63 (95% CI, 0.43–0.80) for AJCC (ΔAUC = + 0.19; DeLong *p* = 0.048). In the NT group, AUC was 0.78 (95% CI, 0.56–0.94) versus 0.58 (95% CI, 0.34–0.79) (ΔAUC = + 0.20; *p* = 0.071) (Table 6).

**Table 6:** Bedside scores versus AJCC (temporal test sets)

Cohort	Sample	Score AUC (95% CI)	AJCC AUC (95% CI)	ΔAUC	DeLong *P*
Up-front Surgery	Development (*n* = 57)	0.84 (0.75–0.92)	0.68 (0.56–0.79)	0.16	0.01
	Test (*n* = 24)	0.82 (0.66–0.95)	0.63 (0.43–0.80)	0.19	0.048
Neoadjuvant therapy	Development (*n* = 36)	0.81 (0.68–0.92)	0.60 (0.44–0.75)	0.21	< 0.01
	Test (*n* = 16)	0.78 (0.56–0.94)	0.58 (0.34–0.79)	0.2	0.07

Head-to-head benchmarking of the bedside scores versus AJCC 8th (yp-AJCC for neoadjuvant therapy) in the held-out temporal test samples. Reported metrics include AUC (95% CI) with paired DeLong tests, and logistic calibration (intercept, slope)

*cNRI* Continuous net reclassification improvement, *IDI*  Integrated discrimination improvement

Calibration in the test samples also remained closer to the ideal 45° reference line for the bedside models than for AJCC (Supplementary File, Figure S6). Across the combined temporal test sets (*n* = 40), both reclassification and absolute discrimination were improved, with cNRI = 0.26 (95% CI, 0.06–0.44) and IDI = 0.07 (95% CI, 0.02–0.13). Calibration parameters were similarly closer to ideal for the bedside models (intercept = 0.02; slope = 0.92) compared with AJCC (intercept = − 0.31; slope = 0.71).

### Robustness and complementary imaging context

Sensitivity analyses confirmed the robustness of the models under multiple scenarios, including complete-case analyses, a shortened 9-month endpoint, and sequential exclusion of individual predictors (Supplementary File, Table S5). Across these tests, discrimination changed minimally (ΔAUC ≤ 0.05) and calibration slopes remained ≥ 0.88, indicating stable model performance.

Exploratory treatment-by-biomarker interactions (Supplementary File, Table S6) revealed comparatively stronger associations for CAR and CEA within the NT pathway and suggested a pathway-specific vascular effect for hepatic-artery contact ≥ 90°, without altering the final score composition. Complementary anatomic variables including peripancreatic infiltration, ductal and parenchymal dimensions, vascular contact, neural-plexus distances, and nodal morphology, provided mechanistic insight into early systemic dissemination but did not consistently persist under multivariable parsimony (Supplementary File, Tables S7–S9). Convergence diagnostics and complete-case evaluations supported limited imputation-related bias and overall model stability.

## Discussion

Our pathway-specific bedside scores—each composed of five preoperative variables—showed strong and well-calibrated performance, with optimism-corrected AUCs of 0.83 in US and 0.80 in NT cohorts. These values exceed the ~ 0.68–0.70 performance range reported for prior CT-only or CA19-9–based nomograms [23]. Clinical separation was also distinct: 12-month recurrence increased from 11% to 81% across favorable-to-poor strata in US and from 10% to 69% in NT, effectively flagging patients unlikely to benefit from surgery alone [16, 24].

The proposed model is built entirely on CT features that are routinely observable during standard pancreatic MDCT interpretation. Tumor size, necrosis, peripancreatic fat stranding, and vascular contact are visually accessible on conventional multiphase acquisitions (arterial and portal‑venous phases, standard slice thickness) without requiring specialized software, radiomics, or machine‑learning infrastructure. These features are already considered in everyday radiology reading; their frequent omission from structured reports does not stem from invisibility or technical difficulty, but from a historical lack of emphasis under traditional guidelines. The present study demonstrates that these readily available signs carry significant prognostic value and therefore merit explicit documentation. Consequently, implementing the bedside score demands no extra equipment, no dedicated scanner protocol, and no advanced training beyond routine radiological expertise. It is ready for immediate integration into real‑world practice.

The final model’s composition—specifically, the retention of qualitative features over most quantitative ductal and parenchymal measures—may reflect several inherent differences between these variable types. Qualitative features are inherently more observer‑dependent than quantitative measurements, which is an important limitation to acknowledge. Quantitative parameters such as the MPD diameter or the MPD‑to‑parenchymal thickness ratio can have a complex and non‑linear relationship with survival, which conventional regression models may not capture reliably [25]. Sandini et al. found that a higher MPD‑to‑parenchymal ratio was paradoxically associated with improved long‑term survival in up-front resected PDAC, suggesting such measures may not directly indicate aggressive disease [26]. Similarly, Takayanagi et al. reported that PDAC without MPD dilatation had more advanced disease and worse prognosis than those with dilatation, further illustrating the inconsistent prognostic direction of simple ductal metrics [27]. In contrast, qualitative features like rim enhancement and intratumoral necrosis have a more direct link to aggressive pathobiology, with a recent meta‑analysis confirming rim enhancement as a reproducible biomarker of poor differentiation and shorter survival [28]. However, we cannot exclude some degree of model instability given the limited events‑per‑variable, which may have also contributed to the exclusion of quantitative predictors.

Independent imaging predictors retained in our final models—tumor size, intratumoral necrosis, peripancreatic fat stranding, and, in the NT cohort, ≥ 90° hepatic-artery contact—align with previous evidence that portal-venous hypodensity, rim or heterogeneous enhancement, peripancreatic fat stranding, and ductal or parenchymal remodeling reflect aggressive tumor biology and occult dissemination [15, 16]. The MPD/PT ratio, which expresses main pancreatic duct caliber relative to residual gland thickness, has been associated with improved overall survival when elevated [29], and our univariable analyses similarly identified ductal and peripancreatic features as significant indicators of early recurrence. Patterns indicative of extrapancreatic neural invasion such as proximity to the gastroduodenal or inferior pancreaticoduodenal arteries and short distances to the celiac or superior mesenteric plexus, have been linked to early dissemination [30, 31]. Although several vascular contact metrics correlated with recurrence risk in univariable analyses, only hepatic-artery contact remained significant in the NT multivariable model, plausibly reflecting chemoselected residual disease along that arterial margin [32]. CT evaluation of marginal nodal involvement remains limited, with reported accuracies below 65% [23], consistent with the exclusion of nodal status from our final parsimonious models. Taken together, the imaging components of the score appear to capture complementary biological dimensions: overall tumor burden (diameter), hypoxic or necrotic remodeling (intratumoral necrosis), peritumoral stromal and inflammatory extension (fat stranding), and chemoresistant peri-arterial disease (hepatic-artery contact).

The CAR outperformed its individual components and other inflammatory composites, consistent with evidence that CAR integrates both acute-phase response and nutritional reserve, reflecting IL-6–driven stromal signaling [33]. CA19-9 lost independent significance after inclusion of CAR, aligning with its known variability in cholestatic states [16, 17], whereas CEA retained prognostic value in the NT cohort, potentially indicating chemoselected, basal-like phenotypes prone to early systemic dissemination [34]. Collectively, these findings underscore the value of coupling tumor-centric CT features with host-response biomarkers to more comprehensively characterize metastatic potential.

Tumor diameter and CAR demonstrated comparable prognostic gradients across both pathways [16, 33]. Formal interaction testing with false discovery rate adjustment revealed no significant effect modification (e.g., diameter × pathway, q = 0.26), indicating broadly consistent predictive effects. Nonetheless, the limited number of recurrence events in the NT cohort (≈ 18) reduces statistical power, and stringent multiplicity control may have obscured moderate interactions.

Validation in larger cohorts incorporating longitudinal imaging and tissue biomarkers is therefore warranted.

Across temporal test sets, the bedside scores outperformed AJCC 8ᵗʰ staging, with ΔAUC gains of + 0.19 in the US cohort and + 0.20 in the NT cohort, alongside improved reclassification (cNRI ≈ 0.26; IDI ≈ 0.07) and favorable calibration (intercept ≈ 0.02, slope ≈ 0.92) [35]. These improvements underscore that, while anatomy-based staging remains essential for assessing operability, it insufficiently captures the underlying biological heterogeneity of PDAC.

Current guidelines increasingly advocate biologic triage, such as recommending neoadjuvant therapy for patients with markedly elevated CA19-9 levels or radiologically suspicious lymph nodes [17]. Integrating an automated, point-based score into structured radiology reporting could extend this approach—flagging high-risk, CAR-elevated or fat-stranding–positive tumors for intensified systemic therapy or clinical trial enrollment, while enabling de-escalated surveillance in low-risk cases. This pathway-specific framework aligns with contemporary management, in which treatment sequencing is progressively tailored to tumor biology [36, 37].

This study draws strength from its coordinated design across two high-volume tertiary centers operating under harmonized MDCT acquisition and reporting protocols, which enhanced reproducibility and reduced institutional variability. The development of separate predictive models for US and NT cohorts helped minimize spectrum bias, while application of bootstrap optimism correction and temporal hold-out testing provided rigorous internal validation that surpasses the methodological depth of several earlier staging evaluations [35, 36]. While a pooled (unified) model would increase effective sample size and statistical power and yield a single parsimonious tool for clinicians, it would also risk conflating two biologically and clinically distinct states—untreated, US patients versus post-therapy, NT patients—whose predictor distributions and predictor–outcome relationships differ. Properly accounting for this heterogeneity would require pathway indicators and multiple pathway×predictor interaction terms, substantially increasing model complexity and the number of parameters to estimate; given our limited events-per-variable this would elevate overfitting risk, impair calibration, and reduce clinical interpretability for each decision pathway. For these reasons we elected pathway-specific models for stability and direct applicability, and propose evaluating pooled models with formal interaction testing in larger, multicenter cohorts where robust estimation is feasible.

Although 72.2% of the initial cohort was excluded due to non‑PDAC histology, this does not reflect selective narrowing by additional eligibility criteria—the analysis included consecutive, unselected PDAC patients meeting only basic requirements. The observed PDAC proportion among Whipple procedures (27.8%) is lower than the 46–60% reported in recent series from leading centers [38], but it closely matches earlier reports from those centers and contemporary data of similar centers in low-middle-income countries [39–41]. Over time, leading centers have increasingly used non‑operative management for benign conditions, raising their PDAC proportion. In low-middle-income settings, where such alternatives are less accessible, Whipple procedures continue to serve a broader diagnostic spectrum.

Nevertheless, inherent limitations should be acknowledged. The retrospective design within a single-geography region may constrain generalizability, and despite rigorous internal validation, confirmation in independent external cohorts remains necessary [42]. We did not perform a formal sample‑size calculation and, being limited to available cases (*n* = 133), the resulting low events‑per‑variable, especially after the 70/30 split, reduced power for exploratory interaction analyses, may have compromised model stability, and increased the risk of overfitting despite internal bootstrap validation and ridge penalization. However, the primary goal of this study was exploratory – to identify which combination of CT and inflammatory features showed the strongest signal for predicting early recurrence and to provide a foundation for larger confirmatory studies. We therefore intentionally included all clinically plausible predictors rather than restricting the model prematurely. The retained predictors should be prioritized for future validation. The models are hypothesis‑generating, and external validation in independent larger cohorts is essential before clinical implementation. Although the Kaplan‑Meier curves show clear risk stratification, the number at risk in high‑risk strata is modest at curve’s tail, limiting precision of long‑term estimates; therefore, our primary analysis focuses on the 12‑month binary endpoint, and the curve’s tail should be interpreted cautiously. Furthermore, logistic regression does not fully exploit the time‑to‑event nature of recurrence, whereas a Cox model would use exact timing. However, the primary clinical question is whether a patient will recur within the first postoperative year—a fixed landmark that directly guides adjuvant therapy and surveillance.

Logistic regression yields a direct 12‑month probability, readily convertible to a bedside point score. Moreover, the logistic‑derived risk strata still produced clear separation in time‑to‑event analyses, confirming that the binary model captures clinically meaningful temporal differences.

Thus, the chosen approach prioritizes practical utility without sacrificing validity. Treatment allocation reflected real-world multidisciplinary decision making rather than a uniform algorithm, introducing the potential for residual selection bias. Moreover, although the NCCN criteria and board membership remained stable over time, the board’s perspectives may have evolved subtly in response to emerging literature, potentially leading to minor temporal heterogeneity in pathway assignment and thereby limiting model performance and generalizability.

Excluding eight patients who died within 30 days may introduce survivorship bias, as these early deaths, although attributed to surgical complications, could be more frequent in patients with aggressive tumor biology. Accordingly, a recent national database study has associated PDAC with an elevated risk of sepsis, thromboembolism, and disseminated intravascular coagulation, lending plausibility to this hypothesis [43]. Consequently, excluding these patients could lead to an underestimation of the true 12-month recurrence rate and an overestimation of model discrimination (AUC). Calibration may be optimized for a lower-risk sample, predictor coefficients could be modestly attenuated, predicted probabilities for high-risk patients may be too low, and clinical utility (decision curve analysis) might be overestimated. Approximately 27% of patients had at least one missing predictor variable; although addressed using appropriate statistical methods, some uncertainty in model estimates may persist. Inflammatory biomarkers are also susceptible to variability across measurement platforms. Furthermore, although CT feature assessment demonstrated acceptable inter reader reproducibility, these estimates were derived from a limited number of experienced readers, and subtle findings—such as peripancreatic fat stranding or borderline arterial involvement—may remain sensitive to observer variability in broader clinical practices, as previously reported in prior studies. Although Fine–Gray analysis produced similar results to the Kaplan–Meier analysis, residual bias from informative censoring in the Kaplan–Meier estimates (e.g., unequal follow‑up or unmeasured competing events) cannot be excluded. Finally, molecular or radiomic correlates were unavailable to biologically validate imaging–inflammation associations. Multicenter prospective studies across diverse scanners, populations, and molecular subtypes are warranted to confirm robustness and clinical scalability.

Future prospective studies should pursue external multicenter validation across diverse scanners, institutions, and patient populations to confirm the robustness and generalizability of these risk scores. Such investigations may also evaluate integration of the bedside score with molecular indicators of minimal residual disease, including postoperative circulating tumor DNA [18], and assess its role within evolving perioperative treatment paradigms, including DNA damage repair-targeted or immunotherapeutic strategies such as poly-ADP-ribose-polymerase maintenance for germline BRCA-mutated PDAC. In parallel, future research may explore whether the incorporation of radiomic features or machine learning–based approaches could further enhance predictive performance. Finally, adaptive recalibration frameworks should be considered to sustain model accuracy as systemic therapies, imaging technologies, and biological profiling continue to advance.

## Conclusions

Combining readily extractable CT features—tumor diameter, necrosis, peripancreatic fat stranding, and peri-arterial contact—with systemic inflammatory indices (CAR, NLR, and CEA in NT) generated concise, bedside-oriented scores that showed good discrimination for 12-month recurrence after resection (AUC 0.83 in US; 0.80 in NT) and performed better than AJCC 8ᵗʰ staging while maintaining acceptable calibration. By differentiating roughly ≤ 11% (favorable) from ≥ 69–81% (poor) short-term recurrence risk, these models may support more individualized counselling, perioperative planning, and surveillance strategies, pending external validation in larger multicenter cohorts. 

## Supplementary Information

Supplementary Material 1.

## Data Availability

The datasets used and/or analysed during the current study are available from the corresponding author on reasonable request.

## Abbreviations

AJCC: American Joint Committee on Cancer
AUC: Area under the receiver operating characteristic curve
CAR: C-reactive protein-to-albumin ratio
CBC: Complete blood count
CEA: Carcinoembryonic antigen
cNRI: Continuous net reclassification improvement
CRP: C-reactive protein
CT: Computed tomography
eCRF: Electronic case report form
ESR: Erythrocyte sedimentation rate
GAR: Globulin-to-albumin ratio
GIST: Gastrointestinal stromal tumor
HR: Hazard ratio
ICC: Intraclass correlation coefficients
IDI: Integrated discrimination improvement
IQR: Interquartile range
LP: Linear predictor
MDCT: Multidetector computed tomography
MPD: Main pancreatic duct
MPD/PT: Main pancreatic duct-to-parenchymal thickness ratio
NCCN: National Comprehensive Cancer Network
NLR: Neutrophil-to-lymphocyte ratio
NT: Neoadjuvant therapy
OR: Odds ratio
PAR: Platelet-to-albumin ratio
PDAC: Pancreatic ductal adenocarcinoma
PV: Portal vein
ROC: Receiver operating characteristic
SD: Standard deviation
SMA: Superior mesenteric artery
SMV: Superior mesenteric vein
US: Up-front surgery
