# Supplementary material for: Preoperative CT and inflammatory index–based risk scores for predicting short-term recurrence following resection of pancreatic ductal adenocarcinoma
Source: BMC Surg. 2026 Jul 1;26:627. doi: 10.1186/s12893-026-03948-3 (PMC13591898; doi:10.1186/s12893-026-03948-3)
Supplement: Supplementary file 1 [file 12893_2026_3948_MOESM1_ESM.docx]

**Supplementary Figure S1**

1 Patient excluded due to a synchronous malignancy within five years

544 Patients underwent Whipple surgery

Analytical cohort (133)

151 Patients with PDAC histology

9 Patients excluded according to preoperative CT:

- Pre-operative CT study was unavailable (5)
- Unresectable disease on retrospective review (4)

Up-front surgery (81)

150 Patients with PDAC histology without synchronous malignancy

8 Patients death due to early post-surgery complication

141 Patients with resectable/borderline PDAC at preoperative CT

393 Patients excluded due to non-PDAC histology:

- Intestinal type adenocarcinoma (197)
- Distal cholangiocarcinoma (86)
- Neuro-endocrine Tumor (38)
- Periampulary adenocarcinoma of indetermined origin (36)
- Solid pseudo-papillary tumor (11)
- IgG4 and Chronic pancreatitis lacking available preoperative pathology (7)
- Intraductal papillary mucinous neoplasm (5)
- Mucinous cyst adenoma/carcinoma (4)
- Serous cyst adenoma/carcinoma (3)
- Gall-bladder carcinoma (1)
- GIST (1)
- Lymphoma (1)
- Pseudocyst (1)
- RCC metastasis (1)
- Adenocarcinoma due to FAP (1)

Neoadjuvant therapy (52)

**Figure S1 Study flow diagram**. Cohort assembly from initial screening (2017–2024) through exclusions to the final analysis set, with stratification by treatment pathway (up-front surgery [US], neoadjuvant therapy [NT])

**Supplementary Figure S2**

**Figure S2 Calibration plots in development cohorts**. Calibration curves for the bedside models in the development cohorts (US: up-front surgery, NT: neoadjuvant therapy). Loess-smoothed observed versus predicted 12-month recurrence probabilities are plotted against the ideal 45° line; calibration slope and intercept are reported alongside optimism-corrected estimates from bootstrap resampling.

**Supplementary Figure S3**

**Figure S3 ROC curves in development cohorts.** Receiver-operating characteristic (ROC) curves for the up-front surgery (US) and neoadjuvant therapy (NT) bedside models in the development cohorts, with AUCs (95% CIs) shown. Insets provide event prevalence and the optimism-corrected AUCs derived from 1,000-sample bootstrap validation.

**Supplementary Figure S4**

**Figure S4 Bootstrap coefficient stability.** Distributions of unpenalized regression coefficients (β) for predictors retained in the final models, estimated from 1000 bootstrap resamples. For each predictor, the sign of the coefficient was positive in all bootstrap resamples, and the mean and standard deviation (SD) are reported. CT structural features (peripancreatic fat stranding, intratumoral necrosis, hepatic‑artery contact) showed stable directionality with low relative variability, whereas inflammatory indices (CAR, NLR, CEA) exhibited larger standard deviations, suggesting greater uncertainty in coefficient magnitude despite consistent positive direction.

**Supplementary Figure S5**

**Figure S5 Individual predicted risks (waterfall plots).** Waterfall plots of individual predicted probabilities of 12-month recurrence within up-front surgery (US) and neoadjuvant therapy (NT) cohorts, ordered from lowest to highest risk.

**Supplementary Figure S6**

**Figure S6 Calibration in temporal test cohorts.** Calibration performance of the bedside models in the held-out temporal test samples for up-front surgery (US) and neoadjuvant therapy (NT). Plots show observed versus predicted risk with accompanying calibration intercepts/slopes.

Supplementary Table S1:

Table S1. Demographic characteristics.

| Variable | Up-front surgery | | | | |  | Neoadjuvant therapy | | | | |
| --- | --- | --- | --- | --- | --- | --- | --- | --- | --- | --- | --- |
|  | **Recurrence (n = 47)** | **No recurrence (n = 34)** | **Groups P** | **HR (95% CI)** | **Univariable P** |  | **Recurrence (n = 23)** | **No recurrence (n = 29)** | **Groups P** | **HR (95% CI)** | **Univariable P** |
| Age, y (mean ± SD) | 66.5 ± 10.8 | 63.8 ± 9.4 | 0.24 | 1.03 (0.99–1.06) | 0.13 |  | 61.0 ± 10.4 | 59.3 ± 10.2 | 0.56 | 1.02 (0.98–1.06) | 0.41 |
| Age ≥ 60 y, n (%) | 36 (76.6) | 23 (67.6) | 0.45 | 1.46 (0.74–2.88) | 0.27 |  | 14 (60.9) | 12 (41.4) | 0.26 | 2.17 (0.93–5.04) | 0.07 |
| Female sex, n (%) | 22 (46.8) | 14 (41.2) | 0.66 | 0.96 (0.54–1.70) | 0.89 |  | 8 (34.8) | 10 (34.5) | 1.00 | 1.21 (0.51–2.85) | 0.67 |

Baseline demographics and clinical descriptors summarized as mean±SD, median (IQR), or n (%), with Welch’s t-, Mann–Whitney U-, or χ² tests as appropriate.

| Variable | CT features | | |  | Variable | Laboratory markers | | | |
| --- | --- | --- | --- | --- | --- | --- | --- | --- | --- |
|  | **Total (n=133)** | **US cohort (n=81)** | **NT cohort (n=52)** |  |  | **Total (n=133)** | **US cohort (n=81)** | | **NT cohort (n=52)** |
| Maximum diameter | **0 (0%)** | **0 (0%)** | **0 (0%)** |  | **WBC count** | **0 (0%)** | **0 (0%)** | | **0 (0%)** |
| Tumor area | **0 (0%)** | **0 (0%)** | **0 (0%)** |  | **Neutrophil count** | **0 (0%)** | **0 (0%)** | | **0 (0%)** |
| Dominant location | **0 (0%)** | **0 (0%)** | **0 (0%)** |  | **Lymphocyte count** | **0 (0%)** | **0 (0%)** | | **0 (0%)** |
| Tumor conspicuity | **0 (0%)** | **0 (0%)** | **0 (0%)** |  | **Platelet count** | **0 (0%)** | **0 (0%)** | | **0 (0%)** |
| Peripancreatic fat stranding | **0 (0%)** | **0 (0%)** | **0 (0%)** |  | **PT** | **2 (1.5%)** | **1 (1.2%)** | | **1 (1.9%)** |
| Common bile duct diameter | **0 (0%)** | **0 (0%)** | **0 (0%)** |  | **PTT** | **2 (1.5%)** | **1 (1.2%)** | | **1 (1.9%)** |
| Main pancreatic duct diameter | **0 (0%)** | **0 (0%)** | **0 (0%)** |  | **Albumin** | **3 (2.3%)** | **1 (1.2%)** | | **2 (3.8%)** |
| Parenchymal thickness at MPD | **1 (0.8%)** | **1 (1.2%)** | **0 (0%)** |  | **Glucose** | **7 (6.8%)** | **4 (4.9%)** | | **3 (5.8%)** |
| Attenuation — unenhanced | **0 (0%)** | **0 (0%)** | **0 (0%)** |  | **ESR** | **10 (7.5%)** | **5 (6.2%)** | | **5 (9.6%)** |
| Attenuation — late arterial | **3 (2.3%)** | **2 (2.5%)** | **1 (1.9%)** |  | **CRP** | **15 (11.3%)** | **10 (12.3%)** | | **5 (9.6%)** |
| Attenuation portal-venous | **4 (3.0%)** | **3 (3.8%)** | **1 (1.9%)** |  | **CA19-9** | **12 (9.0%)** | **7 (5.3%)** | | **5 (9.6%)** |
| Attenuation — delayed | **6 (4.5%)** | **4 (4.9%)** | **2 (3.8%)** |  | **CEA** | **14 (10.5%)** | **8 (9.9%)** | | **6 (11.5%)** |
| ΔHU (late arterial) | **0 (0%)** | **0 (0%)** | **0 (0%)** |  | **CA 125** | **11 (8.3%)** | **6 (7.4%)** | | **5 (9.6%)** |
| Tumor necrosis | **0 (0%)** | **0 (0%)** | **0 (0%)** |  | **CA 19-9** | **14 (10.5%)** | **10 (12.3)** | | **4 (7.7%)** |
| Enhancement pattern | **0 (0%)** | **0 (0%)** | **0 (0%)** |  |  |  |  | |  |
| Rim enhancement (late arterial) | **1 (0.8%)** | **0 (0%)** | **1 (1.9%)** |  |  |  |  | |  |
| Adjacent-organ invasion | **0 (0%)** | **0 (0%)** | **0 (0%)** |  |  |  |  | |  |
| Perpendicular diameter | **0 (0%)** | **0 (0%)** | **0 (0%)** |  |  |  |  | |  |
| Minimum arterial distance | **0 (0%)** | **0 (0%)** | **0 (0%)** |  |  |  |  |  | |
| Vascular contact portal vein | **1 (0.8%)** | **1 (1.2%)** | **0 (0%)** |  |  |  |  | |  |
| Vascular contact superior mesenteric vein | **0 (0%)** | **0 (0%)** | **0 (0%)** |  |  |  |  | |  |
| Vascular contact superior mesenteric artery | **0 (0%)** | **0 (0%)** | **0 (0%)** |  |  |  |  | |  |
| Superior mesenteric vein-portal vein confluence | **1 (0.8%)** | **1 (1.2%)** | **0 (0%)** |  |  |  |  | |  |
| Coeliac axis | **0 (0%)** | **0 (0%)** | **0 (0%)** |  |  |  |  | |  |
| Hepatic artery | **0 (0%)** | **0 (0%)** | **0 (0%)** |  |  |  |  | |  |
| Inferior pancreaticoduodenal artery | **0 (0%)** | **0 (0%)** | **0 (0%)** |  |  |  |  | |  |
| superior mesenteric artery -branch only | **0 (0%)** | **0 (0%)** | **0 (0%)** |  |  |  |  | |  |
| Plexus distance (superior mesenteric artery) | **1 (0.8%)** | **0 (0%)** | **1 (1.9%)** |  |  |  |  | |  |
| Plexus distance (coeliac) | **2 (1.5%)** | **2 (2.5%)** | **0 (0%)** |  |  |  |  | |  |

Supplementary Table S2:

Table S2. Frequency of Missing Values in the Total Cohort and by Treatment Pathway

Supplementary Table S3:

Table S3. Characteristics of excluded patients who died within 30 days after surgery.

| Variable | Up-front surgery | | | | | Neoadjuvant therapy | | | | | |
| --- | --- | --- | --- | --- | --- | --- | --- | --- | --- | --- | --- |
|  | **Patient 1** | **Patient 2** | **Patient 3** | **Patient 4** |  | | **Patient 1** | **Patient 2** | **Patient 3** | **Patient 4** | |
| Immediate cause of death | Sepsis | Cerebrovascular accident | Sepsis | Sepsis |  | | Sepsis | Pulmonary embolism | Disseminated intravascular coagulation | Cerebrovascular accident | |
| Interval from surgery to death (days) | 11 | 18 | 8 | 21 |  | | 13 | 5 | 19 | 3 | |
| Maximum diameter, mm | 31 | 28 | 26 | 34 |  | | 40 | 19 | 27 | 28 | |
| Tumor area, mm² | 813 | 709 | 577 | 1072 |  | | 1270 | 544 | 771 | 529 | |
| Dominant location | Head | Head + uncinate | Head | Head + neck |  | | Head + uncinate | Head | Head + uncinate | Head + uncinate | |
| Tumor conspicuity | Poor | Moderate | Poor | Poor |  | | Moderate | Well | Well | Poor | |
| Peripancreatic fat stranding | Negative | Negative | Negative | Negative |  | | Positive | Negative | Negative | Negative | |
| Common bile duct diameter, mm | 8 | 15 | 14 | 15 |  | | 17 | 8 | 9 | 13 | |
| Main pancreatic duct diameter, mm | 1 | 4 | 3 | 6.5 |  | | 7.5 | 0.5 | 5 | 4 | |
| Parenchymal thickness at MPD, mm | 11 | 21 | 20 | 10 |  | | 13 | 12 | 20 | 17 | |
| Attenuation — unenhanced | Iso-attenuating | Hypodense | Iso-attenuating | Hypodense |  | | Iso-attenuating | Iso-attenuating | Iso-attenuating | Iso-attenuating | |
| Attenuation — late arterial | Iso-attenuating | Hypodense | Iso-attenuating | Hypodense |  | | Hypodense | Hypodense | Hypodense | Hypodense | |
| Attenuation portal-venous | Iso-attenuating | Hypodense | Iso-attenuating | Hypodense |  | | Hypodense | Hypodense | Hypodense | Hypodense | |
| Attenuation — delayed | Iso-attenuating | Hypodense | Iso-attenuating | Hypodense |  | | Hypodense | Iso-attenuating | Iso-attenuating | Iso-attenuating | |
| ΔHU (late arterial) | 0 | 10 | 0 | 28 |  | | 40 | 10 | 24 | 45 | |
| Tumor necrosis | Negative | Positive | Negative | Positive |  | | Positive | Negative | Negative | Negative | |
| Enhancement pattern | Homogeneous | Poor | Homogeneous | Homogeneous |  | | Rim | Poor | Homogeneous | | Homogeneous |
| Rim enhancement (late arterial) | Negative | Negative | Negative | Negative |  | | Positive | Negative | Negative | Negative | |
| Adjacent-organ invasion | Negative | Positive | Negative | Positive |  | | Positive | Negative | Negative | Positive | |
| WBC count (×10⁹ L⁻¹) | 7.4 | 3.3 | 9.0 | 8.3 |  | | 8.1 | 4.9 | 7.4 | 6.5 | |
| Neutrophil count (%) | 54.4 | 61.7 | 67.7 | 87.6 |  | | 76.4 | 45.5 | 56.9 | 55.6 | |
| Lymphocyte count (%) | 35.9 | 32.0 | 27.1 | 9.9 |  | | 14.6 | 42.1 | 32.7 | 37.0 | |
| Platelet count (×10⁹ L⁻¹) | 221 | 105 | 263 | 57 |  | | 216 | 193 | 259 | 248 | |
| Albumin (g dL⁻¹) | 3.9 | 3.2 | 3.6 | 3.0 |  | | 3.2 | 3.7 | 2.9 | 3.9 | |
| Glucose (mg dL⁻¹) | 200 | 256 | 171 | 90 |  | | 306 | - | - | 233 | |
| ESR (mm h⁻¹) | 10 | 34 | 19 | 84 |  | | 10 | 20 | 89 | 91 | |
| CRP (mg L⁻¹) | 5 | 10 | 5.5 | 74 |  | | 23.7 | 23 | 25 | 40 | |
| CA19-9 (U mL⁻¹) | 20.1 | 40.3 | 20.6 | 19.5 |  | | 553.2 | 55.0 | 47.8 | 115.2 | |
| CEA (ng mL⁻¹) | 1.5 | 1.5 | 2.2 | 3.8 |  | | 6.2 | 1.5 | 2.5 | 3.2 | |

CRP: C-Reactive Protein; CEA: carcinoembryonic antigen; ESR: erythrocyte sedimentation rate; WBC: white blood cell.

Supplementary Table S4:

Table S4. Predictive performance of cohort-specific scores.

| **Cohort** | **Apparent AUC (95% CI)** | **Optimism-corrected AUC (95% CI)** | **Apparent calibration slope** | **Optimism-corrected slope** | **Calibration intercept** | **Brier score** |
| --- | --- | --- | --- | --- | --- | --- |
| Up-front surgery | 0.86 (0.78–0.93) | 0.84 (0.75–0.92) | 1.00 | 0.94 | 0.01 | 0.17 |
| Neoadjuvant therapy | 0.83 (0.71–0.92) | 0.81 (0.68–0.92) | 0.98 | 0.92 | 0.00 | 0.19 |

Apparent and optimism-corrected discrimination and calibration in development cohorts: AUC (95% CI), calibration slope and intercept, and Brier score. Optimism correction via 1,000-sample bootstrap. Slopes near 1.0 and low optimism support model stability.

Supplementary Table S5:

Table S5. Sensitivity analyses (summary of pre-specified scenarios)

| **Scenario** | **Pathway** | **AUC** | **ΔAUC vs primary** | **Calibration slope** |
| --- | --- | --- | --- | --- |
| Complete-case only | US | 0.83 | −0.01 | 0.95 |
| Complete-case only | NT | 0.80 | −0.01 | 0.92 |
| 9-month endpoint | US | 0.82 | −0.02 | 0.90 |
| 9-month endpoint | NT | 0.79 | −0.02 | 0.88 |
| CAR threshold shift (≥2) | US | 0.83 | 0.00 | 0.94 |
| CAR threshold shift (≥2) | NT | 0.80 | −0.01 | 0.91 |
| Remove fat stranding | US | 0.79 | −0.05 | 0.88 |
| Remove tumor necrosis | US | 0.80 | −0.04 | 0.89 |
| Remove hepatic-artery ≥90° | NT | 0.76 | −0.05 | 0.86 |
| Remove CEA | NT | 0.78 | −0.03 | 0.90 |
| Remove NLR | US | 0.82 | −0.02 | 0.93 |
| Remove NLR | NT | 0.79 | −0.02 | 0.90 |
| Remove CAR | US | 0.81 | −0.03 | 0.91 |
| Remove CAR | NT | 0.77 | −0.04 | 0.88 |

Prespecified sensitivity checks including complete-case models, 9-month endpoint, CAR threshold shift (≥2), and leave-one-predictor-out analyses. For each scenario, the table reports AUC and calibration slope (development) and ΔAUC versus the primary models, confirming robustness of findings.

Supplementary Table S6:

Table S6. Treatment-by-biomarker interaction terms

| **Interaction term (Pathway × Predictor)** | **βinteraction** | **OR interaction (95% CI)** | **Wald P** | **q (FDR)** |
| --- | --- | --- | --- | --- |
| NT × log₂-CAR | 0.33 | 1.39 (1.01–1.92) | **0.04** | 0.12 |
| NT × log₂-NLR | 0.12 | 1.13 (0.86–1.48) | 0.37 | 0.49 |
| NT × log₂-CEA | 0.41 | 1.51 (1.05–2.18) | **0.03** | 0.10 |
| NT × Hepatic-artery contact ≥90° | 0.58 | 1.79 (1.02–3.15) | **0.04** | 0.12 |
| NT × Tumour necrosis (yes) | −0.67 | 0.51 (0.26–1.01) | 0.053 | 0.13 |
| NT × Fat stranding (yes) | −0.54 | 0.58 (0.28–1.19) | 0.14 | 0.23 |

Exploratory multiplicative interaction terms between treatment pathway (US vs NT) and selected biomarkers/predictors for 12-month recurrence. Parameter estimates, standard errors, Wald P values, and notes on false-discovery-rate adjustments are provided as in the original table.

Supplementary Table S7:

Table S7. Peripancreatic infiltration, ductal dimensions, and adjacent-organ invasion.

| **Variable** | **Up-front surgery** | | | | |  | **Neoadjuvant therapy** | | | | |
| --- | --- | --- | --- | --- | --- | --- | --- | --- | --- | --- | --- |
|  | **Recurrence (n = 47)** | **No recurrence (n = 34)** | **Groups P** | **HR (95% CI)** | **Univ. P** |  | **Recurrence (n = 23)** | **No recurrence (n = 29)** | **Groups P** | **HR (95% CI)** | **Univ. P** |
| Peripancreatic fat stranding, n (%) | 26 (55.3) | 4 (11.8) | **<0.01** | 3.42 (1.91–6.15) | <0.001 |  | 15 (65.2) | 4 (13.8) | <0.001 | 6.97 (2.89–16.78) | **<0.01** |
| Common bile duct diameter, mm (mean ± SD) | 12.12 ± 4.59 | 12.16 ± 4.45 | 0.96 | 1.02 (0.95–1.09) | 0.585 |  | 13.89 ± 5.74 | 11.48 ± 3.97 | 0.080 | 1.10 (1.02–1.19) | **0.02** |
| Main pancreatic duct diameter, mm | 3.78 ± 2.42 | 5.01 ± 3.19 | 0.052 | 0.90 (0.81–1.00) | 0.058 |  | 4.26 ± 2.26 | 4.67 ± 2.36 | 0.523 | 0.94 (0.78–1.13) | 0.50 |
| Parenchymal thickness at MPD, mm | 17.38 ± 10.21 | 17.01 ± 4.15 | 0.82 | 1.02 (0.98–1.06) | 0.252 |  | 16.54 ± 6.09 | 14.40 ± 3.71 | 0.147 | 1.08 (0.99–1.17) | 0.09 |
| MPD/parenchyma thickness ratio, % | 25.12 ± 16.80 | 32.25 ± 21.58 | 0.10 | 0.98 (0.97–1.00) | 0.047 |  | 28.32 ± 17.75 | 34.73 ± 19.23 | 0.223 | 0.99 (0.96–1.01) | 0.22 |
| Adjacent-organ invasion, n (%) | 34 (72.3) | 15 (44.1) | **0.01** | 2.22 (1.17–4.23) | 0.015 |  | 15 (65.2) | 19 (65.5) | 1.000 | 0.91 (0.39–2.14) | 0.83 |

Univariable associations for peritumoural features (e.g., peripancreatic fat stranding), ductal measures (MPD calibre, common bile duct), MPD/parenchymal-thickness ratio (MPD/PT), and adjacent-organ invasion. HRs (95% CIs) for recurrence-free survival are shown.

Supplementary Table S8:

Table S8. Vascular contact and neural-plexus distance

|  |  | **Up-front surgery** | | |  | **Neoadjuvant therapy** | | |
| --- | --- | --- | --- | --- | --- | --- | --- | --- |
| **Vessel / plexus variable** | **Category** | **Recurrence n/N (%)** | **HR (95% CI)** | **P** |  | **Recurrence n/N (%)** | **HR (95% CI)** | **P** |
| Portal vein | None | 37/68 (54.4) | Reference | – |  | 16/43 (37.2) | Reference | – |
|  | 90–180° | 7/8 (87.5) | 4.57 (1.91–10.91) | 0.001 |  | 5/6 (83.3) | 3.26 (1.17–9.10) | 0.024 |
|  | 180–270° | 3/3 (100) | 4.89 (1.44–16.64) | 0.011 |  | 2/2 (100) | 9.82 (1.91–50.4) | 0.006 |
|  | 270–360° | 0/2 | — | — |  |  |  |  |
| SMV | None | 34/62 (54.8) | Reference | – |  | 12/28 (42.9) | Reference | – |
|  | 0–90° | 1/1 (100) | 20.29 (2.23–176.9) | 0.006 |  |  |  |  |
|  | 90–180° | 2/8 (25.0) | 0.50 (0.12–2.07) | 0.337 |  | 4/8 (50.0) | 1.10 (0.35–3.41) | 0.870 |
|  | 180–270° | 3/3 (100) | 4.37 (1.28–14.91) | 0.019 |  | 2/5 (40.0) | 1.38 (0.31–6.18) | 0.674 |
|  | 270–360° | 7/7 (100) | 3.63 (1.54–8.59) | 0.003 |  | 5/5 (100) | 2.73 (0.95–7.82) | 0.061 |
| SMV–PV confluence | None | 32/61 (52.5) | Reference | – |  |  |  |  |
|  | 90–180° | 10/13 (76.9) | 1.34 (0.65–2.73) | 0.426 |  |  |  |  |
|  | 180–270° | 4/4 (100) | 4.78 (1.63–14.04) | 0.004 |  | 5/7 (71.4) | 2.61 (0.94–7.28) | 0.066 |
|  | 270–360° | 1/3 (33.3) | 0.55 (0.07–3.99) | 0.550 |  |  |  |  |
| Isolated SMV-branch involvement | Present | 9/11 (81.8) | 1.21 (0.59–2.51) | 0.604 |  | 0/1 | — | — |
| Any venous criterion met† | Present | 12/24 (50.0) | 0.83 (0.43–1.60) | 0.583 |  | 6/15 (40.0) | 0.47 (0.28–1.80) | 0.472 |
| SMA | None | 40/79 (50.6) | Reference | – |  |  |  |  |
|  | 0–90° | 10/13 (76.9) | 1.48 (0.73–3.01) | 0.282 |  |  |  |  |
|  | 90–180° | 5/6 (83.3) | 3.34 (1.26–8.86) | 0.015 |  | 6/10 (60.0) | 2.41 (0.92–6.28) | 0.072 |
| Coeliac axis | 0–90° only | 0/2 | — | — |  |  |  |  |
| Hepatic artery | 0–90° | 1/5 (20.0) | 0.23 (0.03–1.70) | 0.152 |  |  |  |  |
| Hepatic artery | 90–180° | 1/1 | 2.55 (0.35–18.9) | 0.357 |  | 6/7 (85.7) | 3.70 (1.43–9.58) | 0.007 |
| GDA contact > 180° | Present | 18/22 (81.8) | 2.91 (1.60–5.30) | <0.001 |  | 10/17 (58.8) | 2.03 (0.89–4.64) | 0.094 |
| Inferior pancreaticoduodenal artery | Present | 5/7 (71.4) | 1.38 (0.55–3.50) | 0.493 |  | 6/11 (54.5) | 1.39 (0.55–3.53) | 0.488 |
| SMA-branch only | Present | 3/3 | 1.69 (0.53–5.48) | 0.378 |  | 3/7 (42.9) | 0.85 (0.25–2.86) | 0.792 |
| Any arterial involvement‡ | Present | 21/25 (84.0) | 3.25 (1.79–5.87) | <0.001 |  | 14/24 (58.3) | 2.57 (1.11–5.97) | 0.028 |
| Plexus distance (coeliac) | < 15 mm | 17/21 (81.0) | Reference | – |  |  |  |  |
|  | 25–34.9 mm | 9/22 (40.9) | 0.37 (0.17–0.84) | 0.017 |  |  |  |  |
|  | ≥ 35 mm | 8/18 (44.4) | 0.40 (0.17–0.93) | 0.033 |  | 3/13 (23.1) | 0.26 (0.07–1.00) | 0.051 |
| Plexus distance (SMA) | < 5 mm | 24/28 (85.7) | Reference | – |  |  |  |  |
|  | 9–17.9 mm | 6/23 (26.1) | 0.22 (0.09–0.53) | 0.001 |  | 2/12 (16.7) | 0.16 (0.04–0.72) | 0.017 |
|  | ≥ 18 mm | 10/29 (34.5) | 0.44 (0.21–0.92) | 0.029 |  | 1/9 (11.1) | 0.09 (0.01–0.69) | 0.021 |

*Univariable associations for circumferential vascular contact (0–360° in 90° bins) with PV/SMV/SMV–PV confluence/SMA/coeliac axis and main branches, and the minimum arterial distance measured across contiguous sections using a consistent tumor-margin definition. HRs (95% CI) for recurrence-free survival.*

Supplementary Table S9:

Table S9. Lymph-node variables

| **Variable** | **Category** | **Up-front surgery** | | |  | **Neoadjuvant therapy** | | |
| --- | --- | --- | --- | --- | --- | --- | --- | --- |
|  |  | **Recurrence n/N (%)** | **HR (95% CI)** | **P** |  | **Recurrence n/N (%)** | **HR (95% CI)** | **P** |
| Pathologic peri-pancreatic LN | Positive | 7/10 (70.0) | 2.13 (0.95–4.78) | 0.07 |  | 10/15 (66.7) | 2.33 (1.01–5.38) | **0.047** |
| Pathologic “in-limit” LN | Positive | 15/24 (62.5) | 1.33 (0.72–2.46) | 0.36 |  | 13/22 (59.1) | 2.14 (0.94–4.91) | 0.07 |
| Suspicious peri-pancreatic LN (CT) | Present | 17/32 (53.1) | 1.73 (0.86–3.48) | 0.13 |  | 3/5 (60.0) | 0.42 (0.11–1.63) | 0.21 |
| Suspicious “in-limit” LN (CT) | Present | 20/32 (62.5) | 1.73 (0.84–3.54) | 0.14 |  | 3/5 (60.0) | 0.36 (0.09–1.38) | 0.13 |

Univariable findings for nodal size and morphology. Pathologic nodes: short-axis >6 mm at porta hepatis or >10 mm elsewhere; suspicious nodes: clustered, central necrosis and round/irregular morphology: “in-limit” basins (peripancreatic, SMA, coeliac, or porta-hepatis). HRs (95% CI) for recurrence-free survival.
